# Educational Approach to Prevent the Burden of Vaccinia Virus Infections in a Bovine Vaccinia Endemic Area in Brazil

**DOI:** 10.3390/pathogens10050511

**Published:** 2021-04-23

**Authors:** Galileu Barbosa Costa, Jaqueline Silva de Oliveira, Michael B. Townsend, William C. Carson, Iara Apolinário Borges, Andrea M. McCollum, Erna Geessien Kroon, Panayampalli Subbian Satheshkumar, Mary G. Reynolds, Yoshinori J. Nakazawa, Giliane de Souza Trindade

**Affiliations:** 1 Departamento de Análise em Saúde e Vigilância de Doenças não Transmissíveis, Secretaria de Vigilância em Saúde, Ministério da Saúde, Brasília 70723-040, Brazil; 2 Laboratório de Vírus, Departamento de Microbiologia, Instituto de Ciências Biológicas, Universidade Federal de Minas Gerais, Belo Horizonte, Minas Gerais 31270-901, Brazil; jaquelinebmedica@hotmail.com (J.S.d.O.); borges2805@gmail.com (I.A.B.); ernagkroon@gmail.com (E.G.K.); 3 Poxvirus and Rabies Branch, Division of High-Consequence Pathogens and Pathology, Centers for Disease Control and Prevention, Atlanta, GA 30333, USA; gbu3@cdc.gov (M.B.T.); ioy8@cdc.gov (W.C.C.); avz4@cdc.gov (A.M.M.); xdv3@cdc.gov (P.S.S.); nzr6@cdc.gov (M.G.R.); inp7@cdc.gov (Y.J.N.)

**Keywords:** Vaccinia virus, bovine vaccinia, dairy practices, public health, prevention, educational

## Abstract

Bovine vaccinia (BV), caused by Vaccinia virus (VACV), is a zoonotic disease characterized by exanthematous lesions on the teats of dairy cows and the hands of milkers, and is an important public health issue in Brazil and South America. BV also results in economic losses to the dairy industry, being a burden to the regions involved in milk production. In the past 20 years, much effort has been made to increase the knowledge regarding BV epidemiology, etiologic agents, and interactions with the hosts and the environment. In the present study, we evaluated milking practices that could be associated with VACV infections in an endemic area in Brazil and proposed an educational tool to help prevent VACV infections. In our survey, 124 individuals (51.7%) from a total of 240 had previously heard of BV, 94 of which knew about it through BV outbreaks. Although most individuals involved in dairy activities (*n* = 85/91) reported having good hygiene practices, only 29.7% used adequate disinfecting products to clean their hands and 39.5% disinfected cows’ teats before and after milking. Furthermore, 46.7% of individuals reported having contact with other farm and domestic animals besides dairy cattle. We also evaluated the presence of IgG and IgM antibodies in the surveyed population. Overall, 6.1% of likely unvaccinated individuals were positive for anti-*Orthopoxvirus* IgG antibodies, and 1.7% of all individuals were positive for IgM antibodies. Based on our findings, we proposed educational materials which target individuals with permanent residence in rural areas (mainly farmers and milkers), providing an overview and basic information about preventive measures against VACV infections that could enhance BV control and prevention efforts, especially for vulnerable populations located in endemic areas.

## 1. Introduction

The Orthopoxvirus (OPV) genus affects multiple species and carries great importance in human and veterinary medicine [1,2,3]. Among the members of this genus that are pathogenic to humans and capable of significant impact on global public health are: *Variola virus* (VARV), the cause of the lethal and terrifying smallpox disease, which impacted humanity and challenged public health [4,5]; *Monkeypox virus* (MPXV), endemic in Africa and the most significant the most significant threat to public health since the eradication of smallpox [6,7]; *Cowpox virus* (CPXV), a zoonotic virus with rodent reservoirs, has an increasing number of human case reports in Europe recently [8,9]; and *Vaccinia virus* (VACV), the primary component of the smallpox vaccine, and responsible for natural human and animal infections in southern Asia and South America [1,2,10].

Forty years after smallpox eradication, the emergence of zoonotic OPVs has increased worldwide [1,2,8,10,11]. In Asia and South America, VACV has been described in recurrent outbreaks of vesiculopustular disease, affecting mainly dairy cattle, buffaloes, and milkers, creating a burden to public health and the dairy economy [2,10]. In Brazil, VACV outbreaks were recorded at the end of the 1990s in the Southeast region of the country, which concentrates the largest number of dairy farms and bovine herds [12]. Furthermore, VACV infections have been reported in other South American countries in recent years [13,14], with outbreaks described in Colombia [15].

VACV infections in humans are usually associated with mild disease. Infected individuals develop maculopapular lesions on the fingers, hands, or forearms [2]. The lesions initially appear as itchy focal points on the affected skin, followed by the appearance of local edema and the formation of vesicles, which tend to ulcerate into pustules, merging into focal areas of inflammation. Systemic symptoms such as fever, headache, myalgia, and lymphadenopathy are also reported [2,16]. Although lesions are usually found on fingers and hands of affected individuals, the spread to other body areas such as face, eyes, mucosa, chest, and genitals have been reported, possibly as a result of autoinoculation [17,18]. A case of progressive VACV infection has recently been documented in an HIV positive patient in Colombia, which draws attention to the severity of VACV disease in immunosuppressed individuals [19].

Although VACV circulation has been reported throughout Brazil, awareness of the disease remains low [2,20]. A recent study involving healthcare professionals in a VACV endemic area revealed that unpreparedness related to management of VACV infections in humans could be associated to the limited knowledge about OPV infections [20]. Healthcare professionals were unaware of clinical manifestations, treatment, and prevention; which is essential information to recognize and manage VACV infections [20].

Educational measures aimed at health professionals proved to be effective in terms of OPV surveillance [21,22]. Community participation in the recognition and reporting of diseases is an interesting approach that helps to increase surveillance, reduce disease spread, and decrease the impact on public health. Furthermore, educational initiatives have been used to improve the knowledge and behavior toward infectious diseases, which can also work as a prevention method. The goal of this study is to describe the basic hygiene measures adopted during the dairy handling practices in an endemic area in Brazil, as well as to understand the seropositivity of anti-OPV IgG and IgM antibodies among individuals with permanent residence in rural areas, also providing evidence to guide preventive and control measures. We further aimed to produce educational materials that could be used as tools to aid in the prevention of VACV infections.

## 2. Results

In our survey, 124 individuals (51.7%) from a total of 240 had previously heard of BV (Table 1), 94 of which (75.8%) became aware of it during outbreaks in their farms. Nineteen individuals (15.3%) heard of BV from farmers, eight (6.4%) heard from milkers, and only one searched about BV on the Internet.

A total of 91 individuals (from 124) reported being involved in milking practices (Table 2). Although most individuals performing dairy activities (93.4%) reported they had good hygiene practices, only 29.7% said they used adequate disinfecting products (such as chlorine solution) to clean their hands and very few of them (2.5%) disinfected cows’ teats before and after milking. Iodine solution was reported to be used only to disinfect cows’ teats but not to disinfect milkers’ hands. Noteworthily, 46.7% of individuals reported having contact with other animals besides dairy cattle, such as horses (141; 58.7%), dogs (112; 46.7%), and cats (30; 12.5%).

Table 3 shows the characteristics of likely unvaccinated individuals (*n* = 126, age < 36 years old) that tested positive for IgG and all participants (*n* = 240) that tested positive for IgM antibodies. All individuals positive by IgM were older than 36 years and would have been vaccinated, but only one had the presence of a smallpox vaccine scar. Regarding sex, the majority (5 of 7) of IgG positive individuals and IgM positive (3 of 4) were male. Most individuals reported contact with bovines and equids. Involvement in milking activities were reported by 72.7% of individuals (among which 4 were IgG positive and 3 were IgM positive), while consumption of raw milk was reported only by two IgG positive individuals. On the other hand, only one IgM positive female reported consuming cheese made from raw milk, while 57.1% of IgG positive individuals reported doing so. Interestingly, three individuals that tested positive for IgG antibodies were affected by a BV outbreak in 2011. The main symptoms reported by all individuals were fever, headache, myalgia, and lymphadenopathy. They also reported the presence of lesions on their fingers and hands. Two individuals reported the illness lasted at least 15 days, while one lasted for 30 days.

## 3. Discussion

In this study, half of the surveyed individuals knew about BV, and even though the disease is endemic in the region, most participants became aware during an outbreak. This finding highlights the need to spread knowledge about BV (and VACV) and its consequences to public health and local dairy economies. It is also important to emphasize that the study population seems to have limited access to Internet, which could restrict their ability to find information regarding BV and VACV infections. Furthermore, very few individuals received information from healthcare or veterinary professionals [20].

We also explored the knowledge regarding hygiene measures that could help prevent VACV infections from a likely source, cattle handling, among individuals who participate in milking. Most individuals reported practicing simple measures to disinfect their hands (water and soap), and few individuals used chlorine and/or iodine solution for disinfection of hands and cows’ teats. A recent study showed that use of devices typically filled with iodine or chlorine solutions after milking may be effective in deactivating VACV particles, which could be protective against VACV infections [23]. Other studies have also shown that hygienic practices are important to reduce the risk of viral infections, highlighting the importance of frequent handwashing and use of different solutions such as water and soap and hypochlorite solutions to prevent Ebola transmission.

To better understand if the individuals included in this study were exposed to VACV or not, we decided to evaluate the presence of anti-OPV IgG and IgM antibodies; the latter would suggest a recent primary exposure to OPVs. As vaccination against smallpox in Brazil was discontinued in 1978, individuals less than 35 years old are unlikely to have been vaccinated against smallpox and have residual IgG antibodies. Our results also showed that 7 individuals less than 35 years old were IgG positive and four participants tested positive for IgM antibodies. In studies of IgM responses to OPXV in non-western populations, we have found between 3 and 5% of individuals produce positive IgM responses in the absence of recent exposure (M. Townsend, unpublished correspondence). Without clear epidemiological link and knowledge of potential higher background, we cannot confirm recent infection by positive IgM results. However, the finding of IgG responses in 6.1% of unvaccinated individuals clearly indicates prior VACV exposure. Indeed, Serro region is known for a high number of VACV outbreaks [2,24,25,26] and our results provide additional evidence of prior and possible recent viral circulation. These findings thereby highlight the need for an active epidemiological surveillance of OPV in the area, especially due to the burden caused by BV in the local dairy economy.

One young individual (14 years old) that reported no milking activities and no contact with farming animals (dairy cattle and horses) tested positive for IgG antibodies. However, the 14-year-old female reported she had contact with domestic dogs, raising the hypothesis that these animals could act as an alternative source of VACV infection in rural areas. Costa and colleagues have described the presence of VACV DNA in urban domestic dogs from Belo Horizonte, Southeast region of Brazil [27]. Although in that study it was not possible to obtain swab from lesions or other clinical samples to attempt VACV isolation, the detection of VACV DNA through molecular assays suggested VACV infection in urban domestic dogs [27]. Moreover, Peres et al. have also detected VACV DNA in dogs from rural areas during a BV outbreak [28]. In this context, it is also worth mentioning that cats are a common vector for CPXV in Europe, human infections being acquired mainly through direct contact with infected domestic cats [8]. Although the 14-year-old female did not report she had contact with domestic cats, this fact may warrant special attention due to the possibility of domestic cats acting as vectors for VACV. Additional studies should investigate the role of domestic cats as vectors for VACV.

VACV has already been documented to spread within households, including through household fomites [29,30]. However, in some cases, the source of the infection is unknown, especially in individuals who did not participate in milking activities. Costa and co-workers have suggested alternative routes of zoonotic VACV infections in the study area, such as the consumption of cheese and raw milk [26]. In addition, the direct contact with other potential VACV hosts such as cats, dogs, and rodents [27,31,32] could also pose a risk for viral transmission to humans.

During a Buffalopox virus (a close variant of VACV) outbreak in Western Maharashtra, India, Gurav and colleagues suggested that individuals with oral lesions could have been infected due to the consumption of raw milk [33]. Additionally, some studies in Brazil have detected viable VACV particles in milk and dairy products [34,35,36]. However, there’s no evidence of oral infections caused by VACV directly associated with the consumption of raw milk, artisanal cheese, or other dairy products to date. Our findings also highlight the need to better investigate alternative routes of zoonotic VACV transmission, as well as the need to improve the knowledge of the population regarding the risks of potential VACV exposure through raw milk and dairy products.

We developed educational materials that target farmers and milkers and provide an overview and basic information about prevention measures against VACV infections that could enhance BV control and prevention efforts, especially for vulnerable populations located in endemic areas. The proposed materials present valuable information that is helpful for people living in rural areas. It is written in English, Portuguese, and Spanish, and was included in this article as supplementary material (Supplement Figures S1–S6). As the occurrence of BV and natural VACV infections have been documented in South American countries [2,13,14,15], the Portuguese and Spanish versions of the educational material will be helpful for healthcare professionals and policy-makers who are not fluent in English. Public health managers could download the educational material in Portuguese and Spanish and distribute to health departments and to dairy farms during field expeditions aiming to inform, educate, and raise awareness regarding BV and VACV infections.

We believe that educating the most affected population (mainly those at high risk) such as, veterinarians, healthcare professionals, and public health personnel about the different aspects of BV could help reduce the burden on public and veterinary health. The educational materials can also stimulate the rural community to participate in the surveillance activities and provide community members with basic knowledge to protect themselves against the VACV disease. Further studies could be planned to measure the impact of the educational materials presented here, including a comprehensive approach that includes access to government services, improving access to PPE and other resources, educational campaigns, and barriers associated with access to information. The proposed educational materials could also help farmers and dairy workers to better understand which variables are the most important to decrease the burden of BV and VACV during day-by-day activities.

## 4. Materials and Methods

### 4.1. Study Area and Population

This study was performed in Serro city (18°36′17″ S 43°22′46″ W), located at 312 km North of Belo Horizonte city, the capital of Minas Gerais State [2,37,38]. According to information from Brazilian Institute of Geography and Statistics (IBGE), the total population estimated for 2012–2013 in Serro is 20,833 inhabitants, distributed in an area of 1,217,813 km^2^, with 7938 inhabitants located in rural areas [37]. BV is endemic in Serro region and different studies have already reported several outbreaks since 2005 [2,24,25,26]. Furthermore, other studies conducted in the absence of outbreaks have also reported VACV infections in humans, dairy cattle, horses, and wild animals through serological assays [23,32,37,38,39].

Serro has a tradition of cheese production, introduced by the Portuguese settlers from Serra da Estrela region more than two centuries ago, when the first cattle farms in the region were formed to support the gold and diamond mining industries [40]. With the decay of the gold cycle, the municipality of Serro intensified its agricultural activity and cheese was the product that guaranteed foreign exchange for the region and the whole state, due to the volume and quality that it represented for the market. Since then, Serro cheese has been featured as a symbol of cultural identity by the peculiar flavor and mode of production (using raw milk) and is recognized as a culturally important and distinct product [41].

### 4.2. Dairy Practices and Exposure Assessment

During 2012–2013, a total of 240 individuals with permanent residence in rural areas were invited to participate in the survey and a structured questionnaire was applied as previously described [37]. The farms were selected based on a list of farms provided by the local health stations. We attempted to include all individuals in the household, and each individual was interviewed separately.

The questionnaire was developed to capture demographic data and daily activities in rural areas of Serro that could be potentially associated with VACV exposure [37]. The questionnaire was administered via face-to-face interviews. The questionnaire consisted of questions, divided into five sections: socio-demographic data, bovine vaccinia knowledge (informative questions about the occurrence of BV outbreaks), information on previous outbreaks, dairy practices, and preventive measures. In this study, we focused on questions related to bovine vaccinia knowledge, dairy practices, and preventive measures. The “knowledge questions” (better described as informative questions regarding BV outbreaks), were designed to assess awareness of the population regarding BV outbreaks or VACV infections. Questions about dairy practices were focused on milking practice and milkings per day, consumption of raw milk and cheese made from raw milk, and manipulation of raw milk for cheese production. Furthermore, the questions related to “hygiene measures” allowed us to assess if the population could somehow avoid viral exposure. We also collected serum samples from interviewees for serologic evidence of VACV exposure.

### 4.3. IgG and IgM Antibodies Detection

Serum samples were screened by an enzyme-linked immunosorbent assay (ELISA) for the presence of anti-OPXV IgG and IgM antibodies as previously described [42] but with a modification for positive identification described below. Individuals aged ≥ 36 years were considered as vaccinated during the smallpox vaccination campaign and a positive result would not be possible to interpret accurately (vaccination vs exposure). We decided to test only individuals younger than 36yo (*n* = 126) for the presence of IgG antibodies, while all 240 individuals were tested for the presence of IgM antibodies.

A purified and formaldehyde-inactivated VACV-Dryvax strain diluted in 0.01 M carbonate buffer (Sigma-Aldrich^®®^, Saint Louis, MO, USA) at pH 9.6 was used as antigen. All samples were tested in duplicate. Specific antibody binding was revealed by the addition of 3,3′,5,5′-tetramethylbenzidine (TMB, Sigma-Aldrich^®®^, Saint Louis, MO, EUA) substrate with absorbance measured at 450 nm. Values were expressed as the optical density (OD). A cut-off value (COV) was determined by the mean of the ODs obtained from the negative controls plus three standard deviations of the mean. This COV was subtracted from OD values resulting in the final corrected OD (OD-COV). An additional criterion of 0.1 above this OD-COV was added to account for additional variation in non-western populations.

### 4.4. Ethical Considerations

Ethical clearance was obtained from Research Ethics Committee of Universidade Federal de Minas Gerais under the registration protocol FR–413704. Prior to data collection, the objectives of the study were explained and an informed consent was obtained from all participants. For minors, consent was obtained from parents or guardians.

## 5. Conclusions

After smallpox eradication, the importance of poxviruses has decreased in human medicine, not being unusual. Our data show that even individuals living in endemic areas at high risk do not know about BV or VACV infections. This study points to the importance of knowledge about BV among farmers and rural workers, which could enhance the efforts for BV control and prevention. Our study also provides valuable insights to health authorities and decision-makers regarding basic preventive measures for BV that may be useful for improving surveillance and response.

## Figures and Tables

**Table 1:** Sources of information regarding bovine vaccinia (BV) reported by participants in rural areas of Serro city, Minas Gerais State, Brazil.

Sources of Information	Number of Participants (%)
Participants Who Had Previously Heard of BV	124/240 (51.7%)
From a farmer	19/124 (15.3%)
From a milker	8/124 (6.4%)
From health care professional	7/124 (5.6%)
From a veterinarian	7/124 (5.6%)
From TV	7/124 (5.6%)
From radio	6/124 (4.8%)
From Internet	1/124 (0.8%)
During an outbreak	94/124 (75.8%)

**Table 2:** Hygiene measures adopted by 91 individuals who reported being involved in milking activities in rural areas of Serro city, Minas Gerais State, Brazil.

Hygiene Measure	*n*
Disinfection of hands (*n* = 85)	
With water and soap only	85/91 (93.4%)
With chlorine solution	27/91 (29.7%)
Time frame	
Before start milking only	1/85 (1.2%)
Between different cows	83/85 (97.6%)
Before start and after finish milking	1/85 (1.2%)
Disinfection of cow’s teats (*n* = 78)	
Water and soap only	78/91 (85.7%)
Chlorine solution	24/91 (26.4%)
Iodine solution	36/91 (39.5%)
Time frame	
Before start milking only	9/78 (11.5%)
Between different cows	67/78 (85.9%)
Before start and after finish milking	2/78 (2.5%)
Disinfection of milking machine *	31/91 (34.1%)
Time frame *	
Before start milking only	0/31
After start milking only	14/31 (45.2%)
Before start and after finish milking	17/31 (54.8%)

* For those who reported mechanical milking only.

**Table 3:** Characteristics of IgG and IgM positive individuals from rural areas in Serro city, Minas Gerais, Brazil.

		Serology		Presence of Smallpo × Vaccination Scar	Contact with Animals				Practice Milking	Raw Milk Consumption	** Cheese Consumption	*** Hygiene Practices		
Gender	Age	IgG OD-COV *	IgM OD-COV *		Bovines	Equids	Dogs	Cats				Disinfection of Hands	Disinfection of Cow’s Teats	Disinfection of Milking Machine
M	32	+ (1.163)	-	No	Yes	Yes	Yes	No	Yes	No	NA	Yes	Yes	No
F	14	+ (1.063)	-	No	No	No	Yes	No	No	No	NA	No	No	No
M	31	+ (0.998)	-	No	Yes	Yes	NA	NA	Yes	No	NA	Yes	Yes	No
F	33	+ (0.991)	-	No	No	No	NA	NA	No	No	Yes	No	No	No
M	21	+ (0.791)	-	No	Yes	Yes	Yes	No	Yes	Yes	Yes	Yes	Yes	No
M	29	+ (0.568)	-	No	Yes	Yes	NA	NA	Yes	No	Yes	Yes	Yes	No
M	53	+ (0.275)	-	No	Yes	Yes	NA	NA	Yes	Yes	Yes	Yes	Yes	No
F	38	-	+ (0.101)	No	Yes	Yes	Yes	No	No	No	Yes	No	No	NA
M	70	-	+ (0.123)	Yes	No	Yes	Yes	No	Yes	No	NA	Yes	Yes	NA
M	67	-	+ (0.472)	No	Yes	Yes	Yes	No	Yes	No	NA	Yes	Yes	NA
M	39	-	+ (0.110)	No	Yes	Yes	NA	NA	Yes	No	NA	Yes	Yes	Yes

* OD-COV (cut-off value), which was determined by the mean of the optical density obtained from the negative controls plus three standard deviations of the mean. ** Cheese made from raw milk. *** For those who reported practicing milking.

## Data Availability

Data is contained within the article or supplementary material.

## Supplementary Materials

The following are available online at https://www.mdpi.com/article/10.3390/pathogens10050511/s1. Figure S1: Quick suggestions, in English, of guides and practices to reduce the burden of bovine vaccinia (BV) among farmers and milkers, Figure S2: Booklet with detailed suggestions, in English, on how to recognize lesions caused by VACV and hygiene measures that can be adopted during the milking process, Figure S3: Quick suggestions, in Spanish, of guides and practices to reduce the burden of bovine vaccinia (BV) among farmers and milkers, Figure S4: Booklet with detailed suggestions, in Spanish, on how to recognize lesions caused by VACV and hygiene measures that can be adopted during the milking process, Figure S5: Quick suggestions, in Portuguese, of guides and practices to reduce the burden of bovine vaccinia (BV) among farmers and milkers, Figure S6: Booklet with detailed suggestions, in Portuguese, on how to recognize lesions caused by VACV and hygiene measures that can be adopted during the milking process.
