# Supplementary material for: Educational Approach to Prevent the Burden of Vaccinia Virus Infections in a Bovine Vaccinia Endemic Area in Brazil
Source: Pathogens. 2021 Apr 23;10(5):511. doi: 10.3390/pathogens10050511 (PMC8145679; doi:10.3390/pathogens10050511)
Supplement: Supplementary file 1 [file pathogens-10-00511-s001.zip › Supplementary figure 1 English.pdf]

DO YOU KNOW BOVINE VACCINIA?

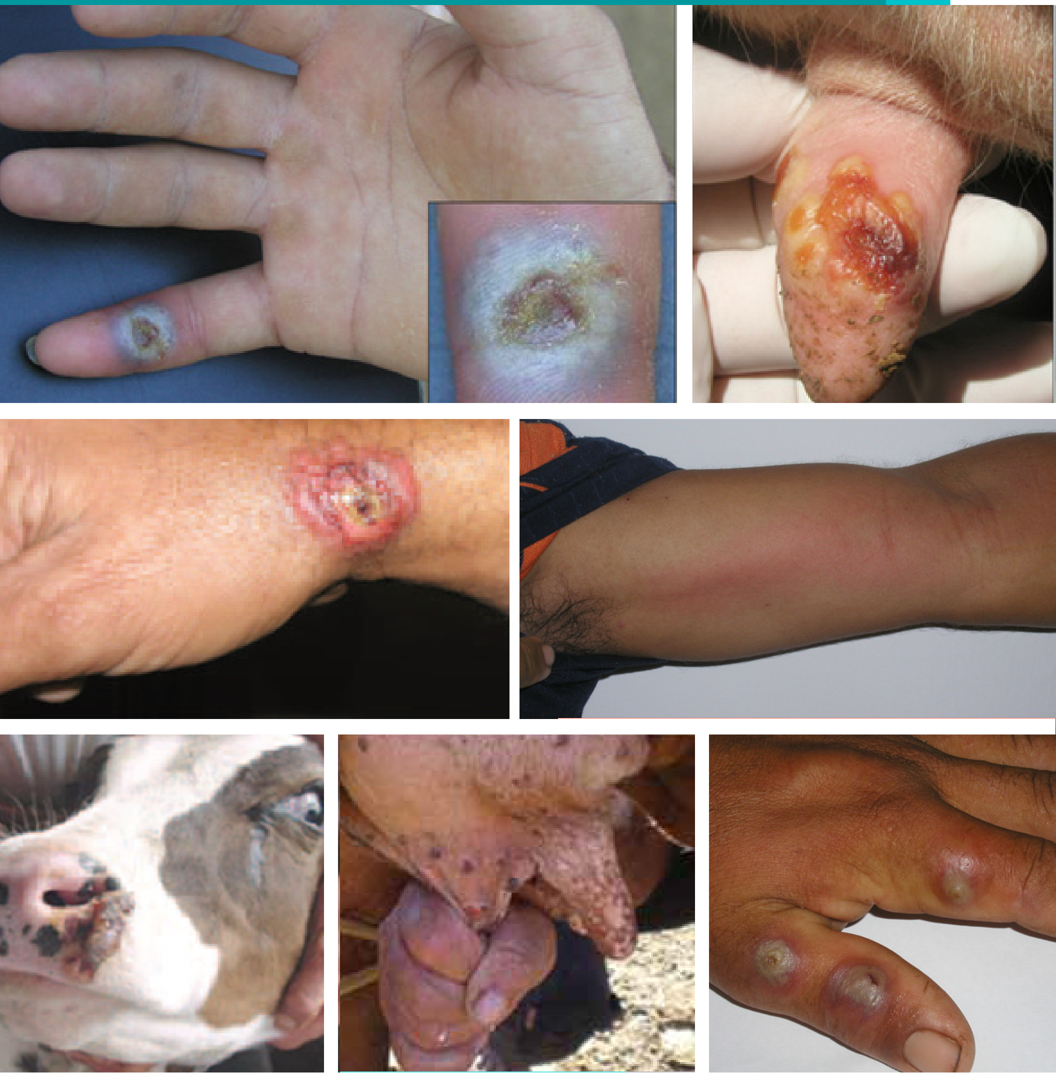

Bovine Vaccinia (BV) is a disease caused by Vaccinia virus (VACV). BV affects mainly dairy cattle. However, other farming animals like horses can also be infected by VACV.

HOW TO PREVENT

Wash your hands with water and soap BEFORE and AFTER milking every cow

Disinfect your gloves (if using any), udders and teats of cows, and the mechanical equipment by using iodine solution or chlorine solution (0,5%) BEFORE and AFTER milking each cow

Use disposable paper towel to dry udders and teats. AVOID using cloth towels, because it can spread the virus easily

Infected animals can shed viral particles in the feces. Keep the barns as clean as possible to reduce the risk of virus spreading

ALWAYS check the udder and teats of cows, and the mouth of calves and horses. TALK to the veterinarian in case you see any lesion

HOW TO IDENTIFY THE DISEASE?

Pimples or bleeding lesions (with or without scabs) can appear in the udder and teats of cows, in the mouth of calves and horses. IT IS RECOMMENDED that milkers use gloves to manipulate animals. Otherwise, milkers can easily get infected

When sick, milkers can get pimples or bleeding lesions mainly on their hands and forearms. Lymphadenopathy, fever, and body ache are also common

If sick, individuals should cover the lesions with loose gauze dressings to avoid transmitting the virus to other individuals, animals, and spreading the virus in the environment

HOW TO TREAT

Clean the udder and teats of cows with water and soap. You can use iodine solution (1-2%) or chlorine solution (0,5%)

Identify the sick animals that present pimples or bleeding lesions and milk them at the end, AFTER milking the healthy cows

In case of any sick animal in the property, keep them quarantined for 20-28 days. Check with a veterinarian for the best adequate treatment

DO NOT SELL OR TRADE SICK COWS. If sick, the animals should be kept isolated in the farm until completely healed to AVOID disease spread in the region

If you have sick animals in your farm, YOU SHOULD CALL the health and veterinarian authorities, so they can take proper care of the animals

In case you or anyone on your farm getting sick, you should immediately seek for a healthcare station and show this card to a healthcare professional

MEDICAL AND VETERINARY DOCTORS

The use of corticosteroids and removing the scabs from the lesions can exacerbate the patients' clinical condition

ECOVIR
UFMG

Source:
Grupo de Pesquisa em Ecologia de Viroses Emergentes (UFMG); Trindade et al., 2003; 2007; Imagens: Leite et al., 2005; Abrahão et al., 2010; Assis et al., 2013.
