# Supplementary material for: Educational Approach to Prevent the Burden of Vaccinia Virus Infections in a Bovine Vaccinia Endemic Area in Brazil
Source: Pathogens. 2021 Apr 23;10(5):511. doi: 10.3390/pathogens10050511 (PMC8145679; doi:10.3390/pathogens10050511)
Supplement: Supplementary file 1 [file pathogens-10-00511-s001.zip › Supplementary figure 2 English.pdf]

TEN TIPS TO RECOGNIZE AND PREVENT BOVINE VACCINIA IN YOUR CATTLE HERD

10

To recognize and control!

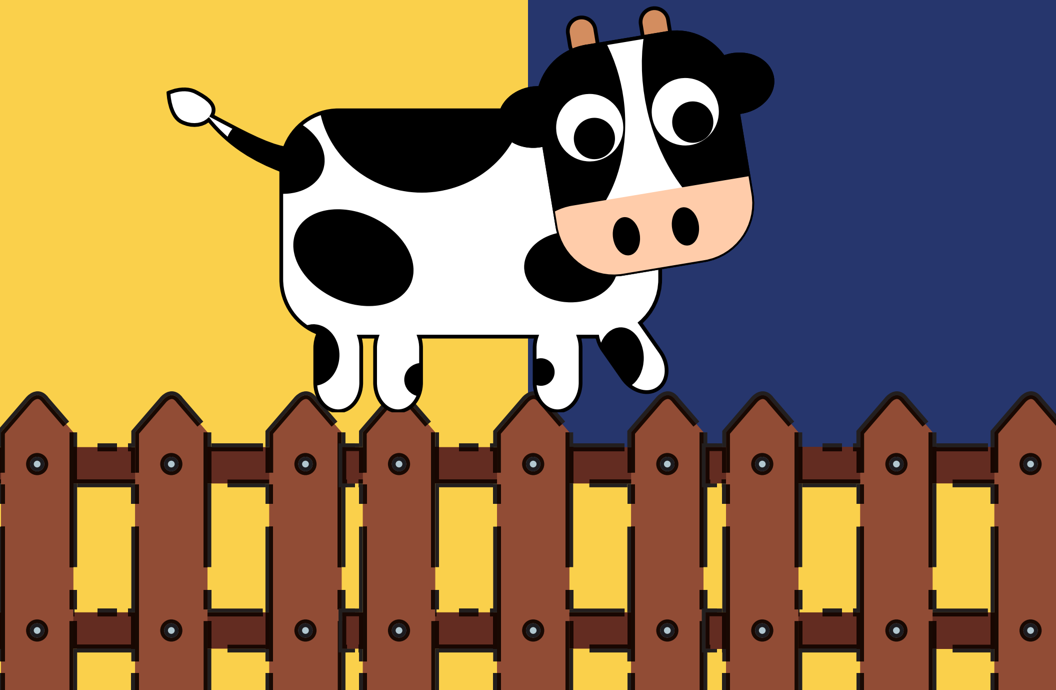

BOVINE VACCINIA (BV) IS A VIRAL DISEASE CAUSED BY VACCINIA VIRUS. BV AFFECTS MAINLY DAIRY CATTLE, DECREASING MILK PRODUCTION, ALSO AFFECTING THE MILKERS WHO HAVE DIRECT CONTACT WITH SICK COWS.

The virus can be transmitted from sick cows to milkers through direct contact with lesions during the milking process. Calves can also get infected when feeding from sick cows. Milkers can also transmit the virus to other healthy cows and other members in the farm through physical contact.

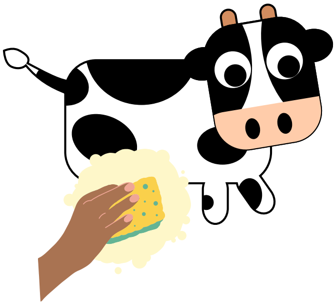

01

WASH THE COWS' TEATS WITH WATER AND SOAP, REMOVING THE RESIDUAL CLAY AND DIRT THAT NORMALLY ACCUMULATES. AVOID SOAKING THE SUPERIOR PART OF THE UDDER (WHERE THE MILK IS NATURALLY STORED), TO AVOIDING THE DIRT REACHING THE TEATS.

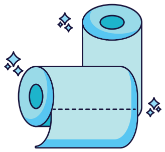

DRY WITH DISPOSABLE PAPER TOWELS

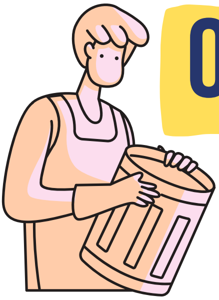

02

DIP EACH TEAT IN IODINE OR CHLORINE SOLUTIONS BEFORE MILKING. THIS PROCESS IS CALLED PRE-DIPPING AND THE OBJECTIVE IS TO COMPLETELY CLEAN THE TEATS, KILLING BACTERIA, FUNGI, AND VIRUSES THAT MAY CAUSE MASTITIS OR OTHER DISEASES. IODINE AND CHLORINE SOLUTIONS CAN BE APPLIED BY USING DISPOSABLE CUPS. IT IS NOT RECOMMENDED TO USE THE REMAINING SOLUTION FROM THE CUPS ON OTHER ANIMALS. BY CHOOSING DISPOSABLE CUPS, YOU CAN GUARANTEE THAT THE SOLUTIONS USED FOR ONE COW WILL NOT BE USED ON OTHERS.

CONSULT MEDICAL OR VETERINARIAN PROFESSIONALS TO DETERMINE THE APPROPRIATE CONCENTRATION OF IODINE AND CHLORINE SOLUTIONS. THE EXCESS OF IODINE OR CHLORINE SOLUTIONS CAN CAUSE LESIONS OR EVEN BURN THE COWS' TEATS.

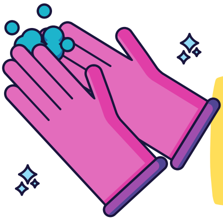

03

WASH YOUR HANDS WITH WATER AND SOAP BEFORE MILKING. IN CASE OF MECHANICAL MILKING, CLEAN THE EQUIPMENT PROPERLY ACCORDING TO THE MANUFACTURING MANUAL BEFORE MILKING.

TAKE CARE OF YOUR MECHANICAL MILKER! BESIDES CLEANING IT DAILY (BEFORE AND AFTER MILKING), IT IS IMPORTANT TO CHANGE THE DISPOSABLE PARTS OF THE MACHINE AS NEEDED TO AVOID ACCUMULATION OF MICROORGANISMS. MAKE SURE THE VACUUM PRESSURE IS ADEQUATE AND CONSTANT. HIGHER, LOWER, OR INCONSTANT VACUUM PRESSURES CAN HURT YOUR CATTLE HERD, FACILITATING THE INFECTION BY MICROORGANISMS THAT CAUSE MASTITS OR BOVINE VACCINIA.

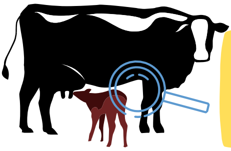

04

CHECK THE COWS' TEATS AND UDDERS, AND THE MOUTH OF THE CALVES DURING MILKING. IF THEY PRESENT PIMPLES OR BLEEDING LESIONS, IT IS RECOMMENDED MILKING THE SICK COWS AT THE END OF THE HERD. PLEASE GO TO THE STEP 10.

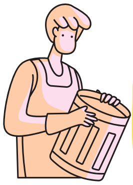

05

AFTER MILKING, MAKE SURE TO DISINFECT THE TEATS DIPPING THEM IN THE IODINE OR CHLORINE SOLUTIONS. GLYCERIN IODINE SOLUTION IS RECOMMENDED DURING THIS STEP CALLED POST-DIPPING. HOWEVER, THE REGULAR IODINE OR CHLORINE SOLUTIONS CAN ALSO BE USED AS LONG AS THEY ARE IN THE ADEQUATE CONCENTRATIONS.

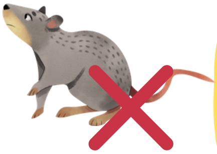

06

CLEAN THE BARN WITH CHLORINE SOLUTION AT THE END OF THE DAY. ALSO, KEEP MILKING AND STORAGE AREAS CLEAN AND FREE OF RODENTS. IT IS ALSO RECOMMENDED TO REMOVE REMAINING FOOD FROM THE BARNS DAILY.

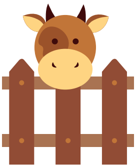

07

IF ACQUIRING NEW FARM ANIMALS (CATTLE OR EQUIDS), KEEP THEM QUARANTINED FOR 20-28 DAYS, FAR FROM THE HERD. CHECK FOR PIMPLES OR LESIONS. IF CATTLE, CHECK THE UDDERS AND TEATS. IF CALVES OR HORSES, CHECK THE MUZZLE AND THE MOUTH.

IF ANY FARM ANIMAL PRESENTS LESIONS OR SIGNS OF BV, KEEP THEM SEPARATED FROM THE HEALTHY HEARD AND PROCEED AS DESCRIBED ON STEP 10.

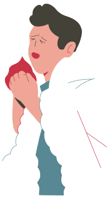

08

ALWAYS CHECK YOUR HANDS AND FOREARMS. IN CASE OF PIMPLES OR LESIONS, YOU SHOULD COVER THEM WITH GAUZE DRESSINGS TO AVOID TRANSMITTING THE VIRUS TO OTHER INDIVIDUALS, ANIMALS, AND SPREADING IT IN THE ENVIRONMENT. IF YOU ALSO PRESENT FEVER, LYMPHADENOPATHY, AND NAUSEAS SEEK MEDICAL HELP. CONTINUE MONITORING FOR SICK ANIMALS AND CALL THE LOCAL HEALTH AUTHORITIES ABOUT THE POSSIBILITY OF BOVINE VACCINIA.

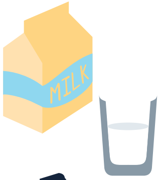

09

ALTHOUGH THERE IS NO SCIENTIFIC EVIDENCE SUPPORTING VIRAL TRANSMISSION THROUGH THE MILK AND ITS DERIVATIVES, IT IS RECOMMENDED TO BOIL THE MILK BEFORE DRINKING IT.

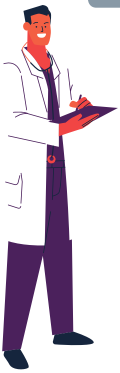

10

IN CASE OF ANY SICK ANIMAL IN THE PROPERTY, CHECK WITH A VETERINARIAN FOR THE BEST ADEQUATE TREATMENT. ALSO, CALL THE LOCAL HEALTH AUTHORITIES TO AVOID SPREADING THE DISEASE IN YOUR HERD AND IN THE REGION.

See below how to identify some lesions compatible with BV

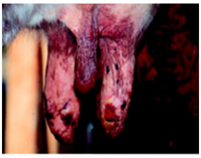

Dairy cows

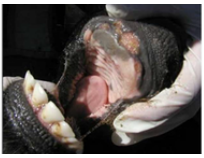

Calves

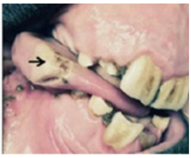

Horses

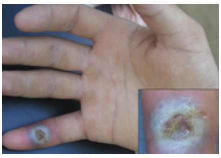

Humans

ECOVIR UFMG

Fonte: Grupo de pesquisa em Ecologia de viroses emergentes, UFMG. Fotos: Trindade et al ., 2003; Assis et al, 2012; Abrahão et al., 2010; 2016.
