# Supplementary material for: Educational Approach to Prevent the Burden of Vaccinia Virus Infections in a Bovine Vaccinia Endemic Area in Brazil
Source: Pathogens. 2021 Apr 23;10(5):511. doi: 10.3390/pathogens10050511 (PMC8145679; doi:10.3390/pathogens10050511)
Supplement: Supplementary file 1 [file pathogens-10-00511-s001.zip › Supplementary figure 3 Spanish.pdf]

¿CONOCES O HAS OÍDO SOBRE LA VACCINIA BOVINA?

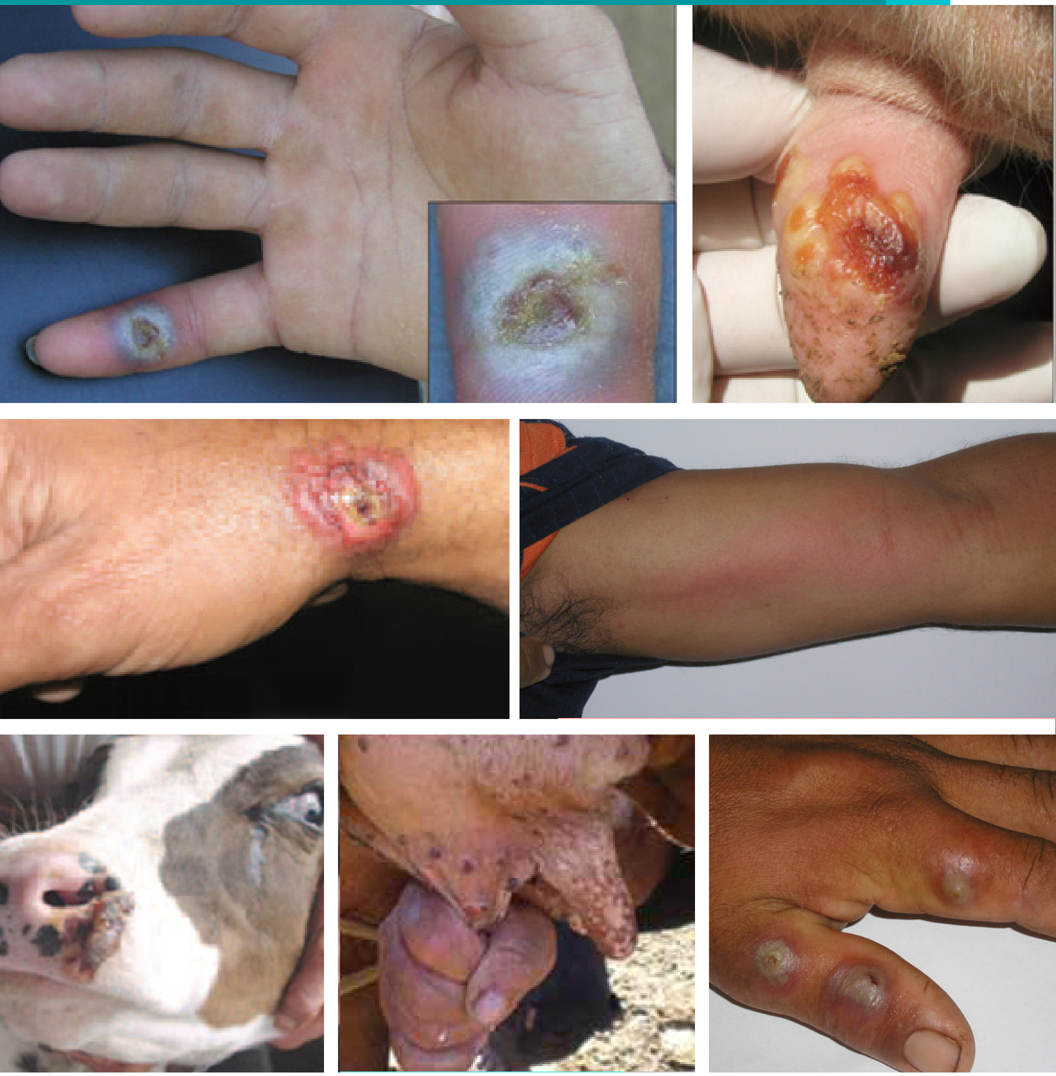

La vaccinia bovina, también conocida como viruela vacuna, es una enfermedad causada por el virus vaccinia. Además del ganado, el hombre y otros animales de granja, como los caballos, también pueden enfermarse.

CÓMO EVITAR

Lávese las manos con agua y jabón ANTES y DESPUÉS DE ordeñar CADA vaca

Desinfecte los guantes (si se usan), las tetillas de las vacas y el equipo de ordeño con glicerina yodada o hipoclorito de sodio al 0.5% ANTES y DESPUÉS DE CADA ordeño

Use toallas de papel desechables para secar los pezones y EVITE el uso de tela, ya que puede facilitar la transmisión del virus

Los animales infectados excretan virus en las heces, así que mantenga el corral limpio para reducir el riesgo de propagación del virus

SIEMPRE observe los pezones de las vacas y la boca de los terneros y caballos, y ALERTE al veterinario si aparece alguna herida

¿CÓMO IDENTIFICAR LA ENFERMEDAD?

Pueden aparecer ampollas o llagas (con o sin corteza) en los pezones de las vacas, en el hocico y / o en la boca de los terneros. Por lo tanto, se RECOMIENDA que la persona que ordeña use guantes para manipular a los animales, de lo contrario puede contraer la enfermedad

Cuando está enfermo, una persona puede tener ampollas o llagas en cualquier parte del cuerpo, especialmente en las manos y los brazos. También son frecuentes los dolores de lengua, corporales, fiebre y cansancio

Cuando están enfermos, las personas deben cubrir las heridas con gasas para evitar transmitir el virus a otras personas, animales y propagar el virus en el medio ambiente

CÓMO TRATAR

Mantenga limpias las heridas de los animales (agua y jabón) y trátelas con una solución de yodo glicerinado (1 a 2%) o hipoclorito de sodio (0,5%)

Identificar vacas enfermas con ampollas o llagas en los pezones y ordeñarlas al final (LÍNEA DE ORDEÑO)

En caso de tener animales enfermos en su propiedad, manténgalo en cuarentena durante 20-28 días. Consulte con un veterinario sobre el tratamiento adecuado

Vender o intercambiar SOLAMENTE animales sanos, las vacas enfermas deben permanecer en la granja hasta que sanen completamente para EVITAR que la enfermedad se propague en la región

Si la enfermedad aparece en la granja, se DEBE avisar al veterinario y a las autoridades sanitarias para que los animales sean tratados y manipulados adecuadamente

Si usted, algún empleado, amigo o familiar parece estar enfermo, busque ayuda inmediata en un centro de salud y MUESTRE ESTA TARJETA al médico

PARA DOCTORES HUMANOS O VETERINARIOS

El uso de corticoides y el desbridamiento de heridas pueden empeorar considerablemente el cuadro clínico provocado por el virus vaccinia

ECOVIR
UFMG

Fuente:
Grupo de Pesquisa em Ecologia de Viroses Emergentes (UFMG); Trindade et al., 2003; 2007; Imagens: Leite et al., 2005; Abrahão et al., 2010; Assis et al., 2013.
