# Supplementary material for: Educational Approach to Prevent the Burden of Vaccinia Virus Infections in a Bovine Vaccinia Endemic Area in Brazil
Source: Pathogens. 2021 Apr 23;10(5):511. doi: 10.3390/pathogens10050511 (PMC8145679; doi:10.3390/pathogens10050511)
Supplement: Supplementary file 1 [file pathogens-10-00511-s001.zip › Supplementary figure 4 Spanish.pdf]

DIEZ CONSEJOS PARA PREVENIR Y RECONOCER LA VACCINIA BOVINA EN SU REBAÑO

10

¡Reconocer para controlar!

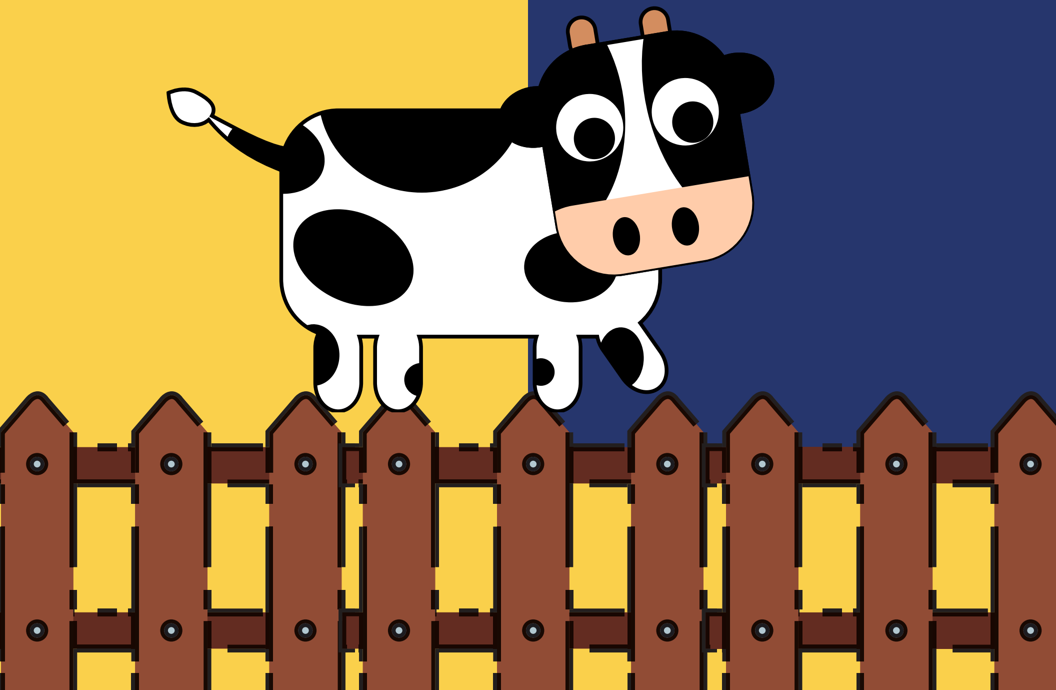

LA VACCINIA BOVINA, TAMBIÉN CONOCIDA COMO VIRUELA VACUNA, ES UNA ENFERMEDAD VIRAL QUE AFECTA PRINCIPALMENTE AL GANADO LECHERO, PROVOCANDO UNA CAÍDA EN LA PRODUCCIÓN DE LECHE Y ENFERMEDAD EN LOS ORDEÑADORES.

Las vacas enfermas transmiten el virus a los ordeñadores que tocan las heridas durante el ordeño y a los terneros que succionan los pezones enfermos. Los ordeñadores, a su vez, pueden transmitir la enfermedad a otros humanos y otros animales.

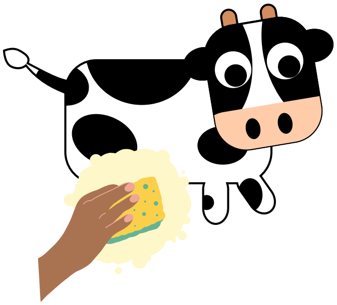

01

LAVAR LOS PEZONES DE LOS ANIMALES CON AGUA Y JABÓN, RETIRANDO EL LODO Y POLVO QUE NORMALMENTE SE ACUMULA EN ESTE LUGAR. EVITE MOJAR LA PARTE SUPERIOR DE LA UBRE (PARTE DONDE SE ALMACENA LA LECHE) PARA QUE LA SUCIEDAD LOCAL NO CAIGA A LOS PEZONES.

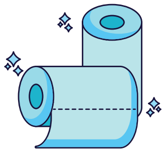

SECAR CON PAPEL DESECHABLE

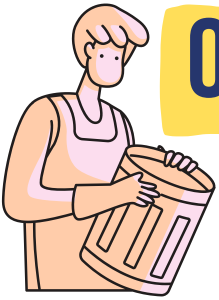

02

INCLUSO ANTES DE ORDEÑAR, SUMERJA CADA PEZÓN EN UNA SOLUCIÓN DE YODO O CLORO. ESTE PROCESO LLAMADO PRE INMERSIÓN SIRVE PARA COMPLETAR LA LIMPIEZA, MATANDO BACTERIAS, HONGOS O VIRUS QUE CAUSAN LA MASTITIS. EL PRODUCTO SE PUEDE APLICAR CON AYUDA DE UN VASO Y EL SOBRANTE NO DEBE REUTILIZARSE EN OTRO ANIMAL. SE PUEDEN ADQUIRIR VASOS ESPECÍFICOS PARA PRE Y POST INMERSIÓN. EN ESTE CASO, OPTE POR COMPRAR VASOS DESECHABLES, ASÍ HABRÁ UNA GARANTÍA DE QUE LA SOLUCIÓN UTILIZADA EN UNA VACA NO SERÁ REUTILIZADA EN LA SIGUIENTE.

PIDA AYUDA A UN VETERINARIO PARA DETERMINAR LAS CONCENTRACIONES DEL PRODUCTO, YA QUE EL EXCESO DE YODO O CLORO PUEDE CAUSAR LESIONES O QUEMADURAS EN LOS PEZONES DE LAS VACAS.

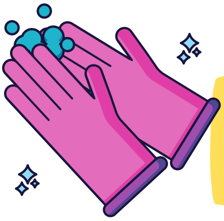

03

ANTES DE ORDEÑAR, LÁVESE LAS MANOS CON AGUA Y JABÓN. EN EL CASO DEL ORDEÑO MECÁNICO, LIMPIAR EL EQUIPO SEGÚN EL MANUAL DEL FABRICANTE Y SÓLO ENTONCES COMENZAR EL ORDEÑO.

CUIDA BIEN TU MÁQUINA DE ORDEÑO, ADEMÁS DE LA LIMPIEZA DIARIA (ANTES Y DESPUÉS DEL ORDEÑO), CAMBIA LAS GOMAS DEL LINER SIEMPRE QUE SEA NECESARIO PARA QUE NO QUEDEN GRIETAS (PUEDEN ALBERGAR MICROORGANISMOS). COMPRUEBE QUE LA PRESIÓN DE VACÍO SEA ADECUADA Y CONSTANTE. LAS PRESIONES DE VACÍO MÁS ALTAS, MÁS BAJAS O CAMBIANTES PUEDEN DAÑAR AL ANIMAL AL FACILITAR LA INFECCIÓN POR MICROORGANISMOS QUE CAUSAN MASTITIS Y/O VACCINIA BOVINA.

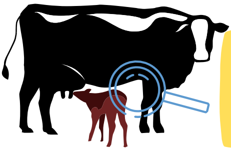

04

REVISAR LOS PEZONES DE LA VACA Y EL HOCICO Y BOCA DEL TERNERO QUE LO ACOMPAÑA. SI HAY AMPOLLAS O LLAGAS (PEQUEÑAS O GRANDES), NO LAS TOQUE SIN GUANTES, DEJE QUE ESTE ANIMAL SEA ORDEÑADO DESPUÉS DE LOS DEMÁS. Y PROCEDA COMO SE DESCRIBE EN EL PUNTO 10.

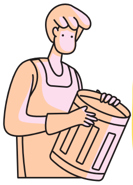

05

DESPUÉS DEL ORDEÑO, DESINFECTE LOS PEZONES NUEVAMENTE SUMERGIÉNDOLOS EN SOLUCIONES DE CLORO O YODO. LA SOLUCIÓN MÁS ADECUADA PARA ESTE PROCESO DENOMINADO "POST INMERSIÓN" ES LA GLICERINA YODADA. SIN EMBARGO, EL CLORO O EL YODO SIMPLE TAMBIÉN SE PUEDEN UTILIZAR CON ÉXITO, SIEMPRE QUE ESTÉN EN LAS CONCENTRACIONES ADECUADAS.

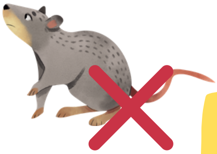

06

AL FINAL DEL DÍA, SE DEBE RASPAR EL ESTIÉRCOL Y LAVAR EL CORRAL CON CLORO O LEJÍA. MANTENGA EL LUGAR DE ORDEÑO Y EL ALMACÉN DE ALIMENTO LIMPIOS Y LIBRES DE ROEDORES. TAMBIÉN SE RECOMIENDA LA ELIMINACIÓN DIARIA DE LOS RESTOS DE COMIDA DE LOS COMEDEROS.

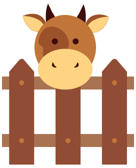

07

SIEMPRE QUE ADQUIERA UN NUEVO ANIMAL (BOVINO O EQUINO). MANTENLO SEPARADO Y ALEJADO DEL RESTO DEL REBAÑO. OBSERVE SI EL ANIMAL TIENE HERIDAS O AMPOLLAS. EN EL CASO DE BOVINOS, OBSERVAR LAS UBRES, EN EL CASO DE TERNEROS O CABALLOS, EXAMINAR LA BOCA Y EL HOCICO.

SI EL ANIMAL MUESTRA SIGNOS DE LA ENFERMEDAD, MANTÉNGALO ALEJADO DE LOS DEMÁS Y PROCEDA COMO SE DESCRIBE EN EL PUNTO 10.

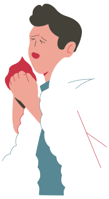

08

SIEMPRE REVISE SUS MANOS Y BRAZOS. SI ENCUENTRA AMPOLLAS Y / O HERIDAS, DEBE CUBRIRLAS CON GASAS PARA EVITAR TRANSMITIR EL VIRUS A OTRAS PERSONAS, ANIMALES Y PROPAGAR EL VIRUS EN EL MEDIO AMBIENTE. SI TIENE FIEBRE, LINFADENOPATÍA Y MALESTAR, CONSULTE A UN MÉDICO. INFORMARLE DE LA EXISTENCIA DE ANIMALES ENFERMOS Y ADVERTIRLE SOBRE LA POSIBILIDAD DE SER VACCINIA BOVINA.

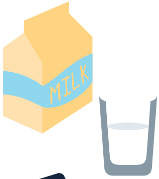

09

AUNQUE LA TRANSMISIÓN DEL VIRUS A TRAVÉS DE LA LECHE AÚN ESTÁ EN ESTUDIO, SE RECOMIENDA HERVIR LA LECHE DE LAS VACAS ANTES DE SU CONSUMO.

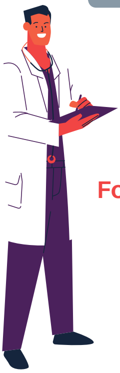

10

EN CASO DE TENER ANIMALES ENFERMOS EN SU PROPIEDAD, CONSULTE CON UN VETERINARIO SOBRE EL TRATAMIENTO ADECUADO. NOTIFIQUE A LAS AUTORIDADES SANITARIAS PARA QUE LA ENFERMEDAD NO SE PROPAGUE EN SU REBAÑO NI EN LA REGIÓN.

Fotografías características de las lesiones para ayudar a identificar la enfermedad

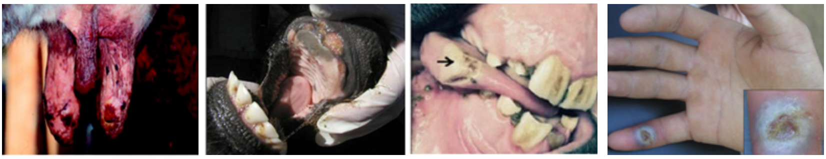

Vaca | Becerro | Caballo | Humano

Fuente: Grupo de pesquisa em Ecologia de viroses emergentes, UFMG. Fotos: Trindade et al., 2003; Assis et al, 2012; Abrahão et al., 2010; 2016.
