# Supplementary material for: Educational Approach to Prevent the Burden of Vaccinia Virus Infections in a Bovine Vaccinia Endemic Area in Brazil
Source: Pathogens. 2021 Apr 23;10(5):511. doi: 10.3390/pathogens10050511 (PMC8145679; doi:10.3390/pathogens10050511)
Supplement: Supplementary file 1 [file pathogens-10-00511-s001.zip › Supplementary figure 5 Portuguese.pdf]

VOCÊ JÁ OUVIU FALAR DA VACCÍNIA BOVINA?

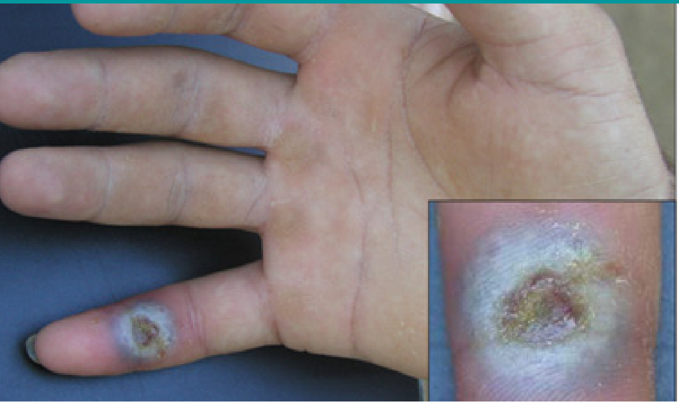

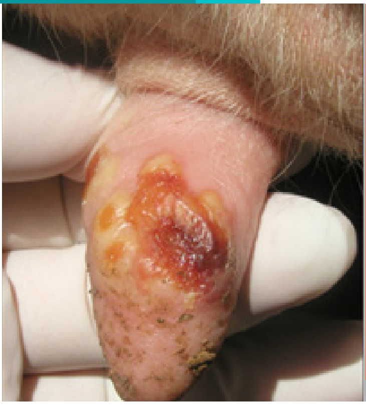

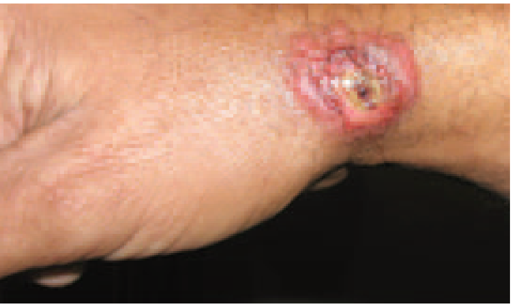

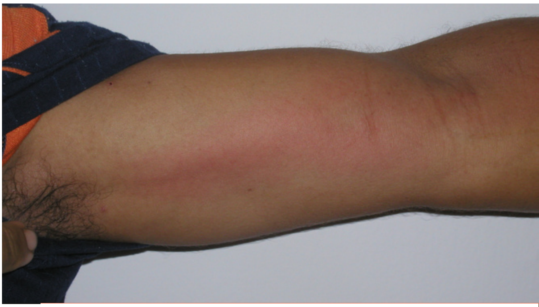

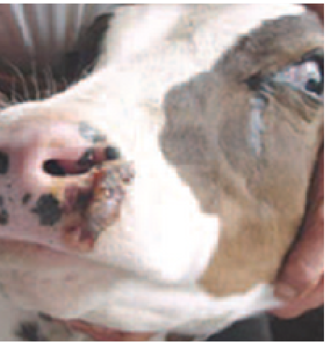

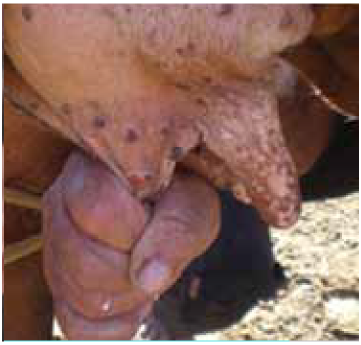

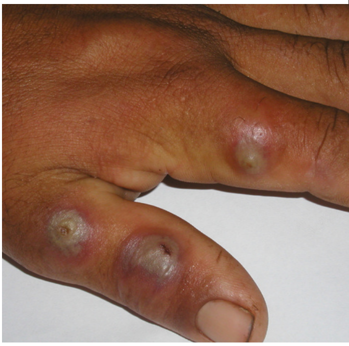

A Vaccínia bovina também conhecida como Varíola bovina é uma doença causada pelo vírus vaccínia. Além do gado, o homem e outros animais da fazenda, como os cavalos, também podem adoecer.

COMO EVITAR

Lavar as mãos com água e sabão ANTES e DEPOIS de CADA vaca ordenhada

Desinfetar as luvas (caso as utilize), as tetas das vacas e o equipamento de ordenha utilizando iodo glicerinado ou hipoclorito de sódio a 0,5% ANTES e DEPOIS de CADA ordenha

Utilizar papel toalha descartável para secar as tetas e EVITAR o uso de pano, pois pode facilitar a transmissão do vírus

Os animais infectados eliminam vírus nas fezes, então mantenha o curral limpo para reduzir o risco de disseminação do vírus

SEMPRE observar as tetas das vacas e a boca dos bezerros e cavalos, e ALERTAR o médico veterinário caso alguma ferida apareça

COMO IDENTIFICAR A DOENÇA?

Bolhas ou feridas (com ou sem casca) podem aparecer nas tetas das vacas, no focinho e/ou na boca dos bezerros. Dessa forma, é RECOMENDADO que o vaqueiro use luvas para manipular os animais, pois do contrário ele pode pegar a doença

Quando doente, o homem pode ter bolhas ou feridas em qualquer parte do corpo, principalmente nas mãos e nos braços. Ínguas, dores no corpo, febre e cansaço também são comum

Quando doente, o indivíduo deve cobrir as lesões com gaze para evitar a transmissão do vírus para outras pessoas, animais e disseminação no meio ambiente

COMO TRATAR

Manter as feridas dos animais limpas (água e sabão) e tratá-las com solução de iodo glicerinado (1 a 2%) ou hipoclorito de sódio (0,5%)

Identificar as vacas doentes apresentando bolhas ou feridas nas tetas e ordenhá-las por último (LINHA DE ORDENHA)

No caso de algum animal doente na propriedade, mantenha-o em quarentena por 20-28 dias. Consulte um veterinário para saber o tratamento mais adequado

Vender ou trocar APENAS os animais curados, os doentes deverão ser mantidos na fazenda até a cura completa para EVITAR que a doença se espalhe na região

Caso a doença apareça na fazenda, o médico veterinário e as autoridades sanitárias DEVEM ser avisadas para que os animais sejam devidamente tratados e manejados

Caso você, algum funcionário, amigo ou familiar aparente estar doente, busque imediatamente ajuda em um posto de saúde e MOSTRE ESTE CARTÃO ao médico

PARA MÉDICOS HUMANOS OU VETERINÁRIOS

O uso de corticosteróides e o debridamento das feridas podem agravar consideravelmente o quadro clínico causado pelo vírus vaccínia

ECOVIR
UFMG

Fonte:
Grupo de Pesquisa em Ecologia de Viroses Emergentes (UFMG); Trindade et al., 2003; 2007; Imagens: Leite et al., 2005; Abrahão et al., 2010; Assis et al., 2013.
