# Supplementary material for: Educational Approach to Prevent the Burden of Vaccinia Virus Infections in a Bovine Vaccinia Endemic Area in Brazil
Source: Pathogens. 2021 Apr 23;10(5):511. doi: 10.3390/pathogens10050511 (PMC8145679; doi:10.3390/pathogens10050511)
Supplement: Supplementary file 1 [file pathogens-10-00511-s001.zip › Supplementary figure 6 Portuguese.pdf]

DEZ DICAS PARA PREVENIR E RECONHECER A VACCÍNIA BOVINA NO SEU REBANHO

10

Reconhecer para controlar!

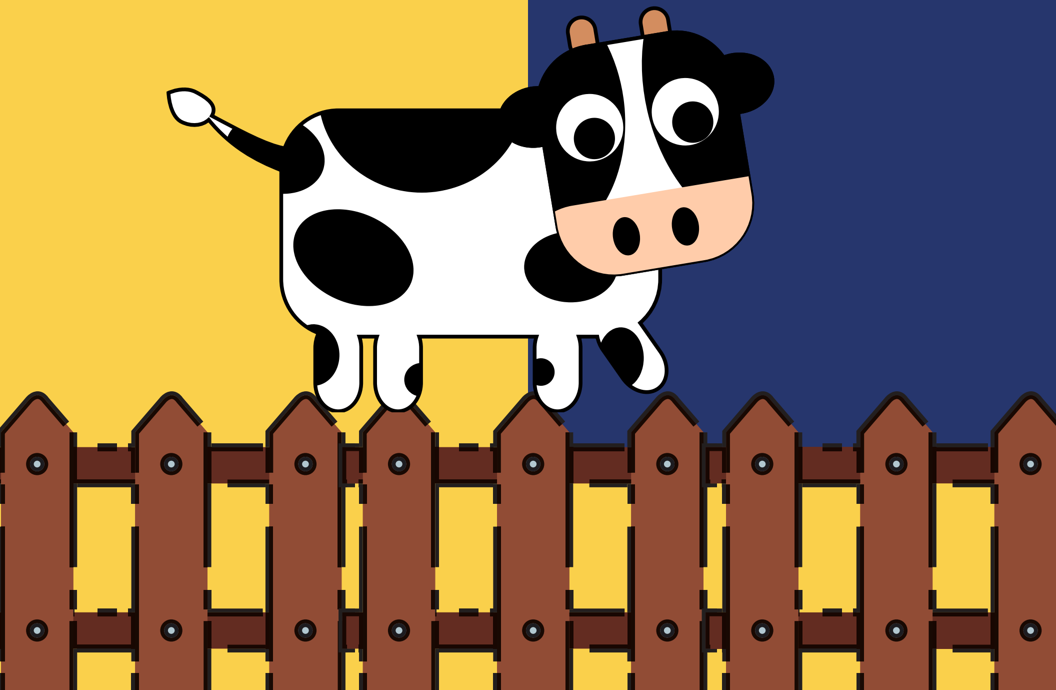

A VACCINIA BOVINA, TAMBÉM CONHECIDA COMO VARÍOLA BOVINA É UMA DOENÇA VIRAL QUE AFETA PRINCIPALMENTE O GADO LEITEIRO, PROVOCANDO QUEDA NA PRODUÇÃO DE LEITE E DOENÇA NOS ORDENHADORES.

As vacas doentes transmitem o vírus para os ordenhadores que encostam nas feridas durante a ordenha e para os bezerros que mamam nas tetas doentes. Os ordenhadores, por sua vez, podem transmitir a doença para outros humanos e outros animais.

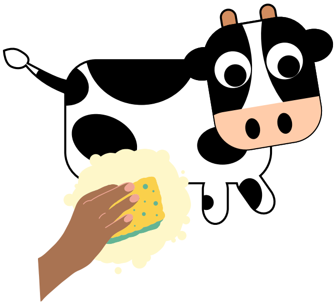

01

LAVE AS TETAS DO ANIMAL COM ÁGUA E SABÃO, RETIRANDO TODO O RESTO DE BARRO E ESTERCO QUE NORMALMENTE SE ACUMULAM NESTE LOCAL. EVITE MOLHAR A PARTE SUPERIOR DO ÚBER (PARTE ONDE O LEITE FICA ARMAZENADO) PARA QUE A SUJEIRA DO LOCAL NÃO ESCORRE PARA AS TETAS.

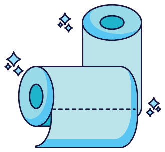

SEQUE COM PAPEL DESCARTÁVEL

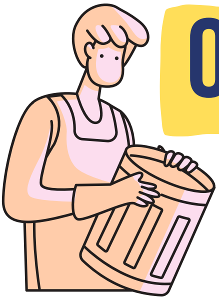

02

AINDA ANTES DA ORDENHA, MERGULHE CADA UMA DAS TETAS EM UMA SOLUÇÃO DE IODO OU CLORO. ESTE PROCESSO CHAMADO DE PRÉ DIPPING SERVE PARA COMPLETAR A LIMPEZA, MATANDO AS BACTÉRIAS, FUNGOS OU VÍRUS CAUSADORES DA MASTITE.
O PRODUTO PODE SER APLICADO COM O AUXÍLIO DE UM COPO E A SOBRA NÃO DEVE SER REAPROVEITADA EM OUTRO ANIMAL.
COPOS ESPECÍFICOS PARA A APLICAÇÃO DE PRÉ E PÓS DIPPING PODEM SER COMPRADOS. NESTE CASO, OPTE PELA COMPRA DE COPOS "SEM RETORNO", ASSIM HAVERÁ GARANTIA DE QUE A SOLUÇÃO UTILIZADA EM UMA VACA NÃO SERÁ RE-UTILIZADA NA PRÓXIMA.

PEÇA AUXILIO A UM MÉDICO VETERINÁRIO PARA DETERMINAR AS CONCENTRAÇÕES DO PRODUTO, POIS O EXCESSO DE IODO OU CLORO PODE CAUSAR LESÕES OU QUEIMADURAS NAS TETAS DAS VACAS.

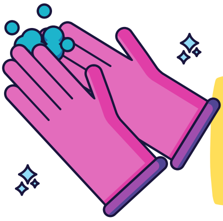

03

ANTES DA ORDENHA, LAVE AS SUAS MÃOS COM ÁGUA E SABÃO. NO CASO DE ORDENHA MECÂNICA, FAÇA A LIMPEZA DO EQUIPAMENTO CONFORME MANUAL DO FABRICANTE E SÓ DEPOIS INICIE A ORDENHA.

CUIDE BEM DA SUA ORDENHADEIRA, ALÉM DA LIMPEZA DIÁRIA (ANTES E DEPOIS DA ORDENHA), TROQUE AS BORRACHAS DA TETEIRA SEMPRE QUE NECESSÁRIO PARA QUE NÃO HAJAM RACHADURAS (PODEM ABRIGAR MICRORGANISMOS). OBSERVE SE A PRESSÃO DO VÁCUO ESTÁ ADEQUADA E CONSTANTE. PRESSÕES DE VÁCUO MAIORES, MENORES OU INCONSTANTES PODEM MACHUCAR O ANIMAL FACILITANDO A INFECÇÃO POR MICRORGANISMOS CAUSADORES DA MASTITE E/OU DA VACCINIA BOVINA.

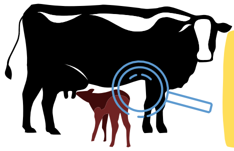

04

OBSERVE AS TETAS DA VACA E O FOCINHO E BOCA DO BEZERRO QUE A ACOMPANHA (SISTEMAS COM "BEZERRO AO PÉ". SEHOUVER BOLHAS OU FERIDAS (PEQUENAS OU GRANDES), NÃO TOQUE-AS SEM LUVAS, DEIXE ESTE ANIMAL PARA SER ORDENHADO DEPOIS DOS OUTROS E PROCEDA COMO DESCRITO NO TÓPICO 10.

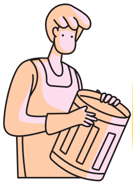

05

APÓS A ORDENHA, DESINFETE NOVAMENTE AS TETAS MERGULHANDO-AS NAS SOLUÇÕES DE CLORO OU IODO. A SOLUÇÃO MAIS ADEQUADA PARA ESSE PROCESSO CHAMADO "PÓS DIPPING" É A DE IODO GLICERINADO. NO ENTANTO, O CLORO OU O IODO SIMPLES TAMBÉM PODEM SER UTILIZADOS COM SUCESSO, DESDE QUE NAS CONCENTRAÇÕES ADEQUADAS.

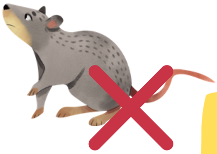

06

AO FINAL DO DIA, O ESTERCO DEVE SER RASPADO E O CURRAL LAVADO COM CLORO (ÁGUA SANITÁRIA). MANTENHA O LOCAL DA ORDENHA E O ARMAZÉM DA RAÇÃO LIMPOS E LIVRES DE ROEDORES. A REMOÇÃO DIÁRIA DAS SOBRAS DE ALIMENTOS NOS COCHOS TAMBÉM É RECOMENDADA.

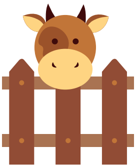

07

SEMPRE QUE ADQUIRIR UM NOVO ANIMAL (BOVINO OU EQUINO) FAÇA A QUARENTENA. MANTENHA-O PRESO E AFASTADO DO SEU REBANHO. OBSERVE SE O ANIMAL APRESENTA FERIDAS OU BOLHAS. NO CASO DE BOVINOS PROCURE NAS TETAS, NO CASO DE BEZERRO OU CAVALOS, EXAMINE A BOCA E FOCINHO.

CASO O ANIMAL APRESENTE SINAIS DA DOENÇA, MANTENHA-O AFASTADO DOS DEMAIS E PROCEDA COMO DESCRITO NO ITEM 10.

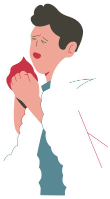

08

OBSERVE SEMPRE SUAS MÃOS E BRAÇOS. CASO ENCONTRE BOLHAS E/OU FERIDAS, DEVE-SE COBRÍ-LAS COM GAZE PARA EVITAR A TRANSMISSÃO DO VÍRUS PARA OUTRAS PESSOAS, ANIMAIS E DISSEMINAÇÃO NO MEIO AMBIENTE. EM CASO DE FEBRE, ÍNGUA NAS AXILAS E MAL ESTAR, PROCURE UM MÉDICO. INFORME-O SOBRE A EXISTÊNCIA DE ANIMAIS DOENTES E ALERTE SOBRE A POSSIBILIDADE DE SER VACCINIA BOVINA.

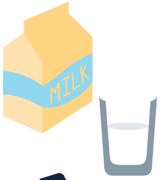

09

EMBORA A TRANSMISSÃO DO VÍRUS PELO LEITE AINDA ESTEJA EM ESTUDO, RECOMENDA-SE QUE O LEITE DAS VACAS DOENTES SEJA FERVIDO ANTES DO CONSUMO.

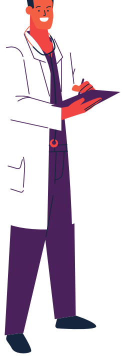

10

QUANDO EXISTIREM ANIMAIS DOENTES CONSULTE O VETERINÁRIO SOBRE O TRATAMENTO ADEQUADO. AVISE ÀS AUTORIDADES SANITÁRIAS PARA QUE A DOENÇA NÃO SE ESPALHE NO SEU REBANHO OU NA REGIÃO.

Fotos características das lesões para ajudar a identificar a doença

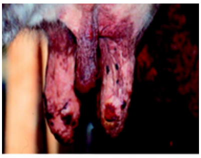
Vaca

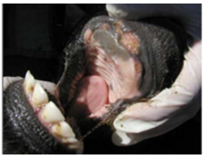
Bezerro

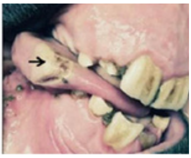
Cavalo

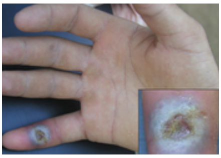
Humano

Fonte: Grupo de pesquisa em Ecologia de viroses emergentes, UFMG. Fotos: Trindade et al ., 2003; Assis et al, 2012; Abrahão et al., 2010; 2016.
